# 

**Published:** 1878-07-20

**Authors:** 


VOL. VIII. JULY 20, 1878. No. 7.

PLEASE PRESENT THE CLAIMS OF THIS JOURNAL TO YOUR MEDICAL BRETHREN.

SHORT COMMUNICATIONS AND PRACTICAL ITEMS SOLICITED.

### THE SOUTHERN MEDICAL RECORD.

A MONTHLY JOURNAL OF PRACTICAL MEDICINE.

**Terms: $2 00 per annum in advance. Club Rates, 5 copies $8.00. Single copies 20 cents.**

*"QUICQUID PRÆCIPIES ESTO BREVIS."*

**ESTABLISHED IN 1870.**

ATLANTA, GA.:

SUNNY SOUTH STEAM PUBLISHING HOUSE.

Address R. C. WORD, M. D., Managing Editor, Atlanta, Ga.

